# Fabrication and Characteristics of a Conductive FeCo@Au Nanowire Alloy for Semiconductor Test Socket Connectors

**DOI:** 10.3390/ma16010381

**Published:** 2022-12-30

**Authors:** In Yea Kim, Jong Won Kim, Byeung Ju Lee, Jae-Hong Lim

**Affiliations:** 1 Department of Materials Science and Engineering, Gachon University, 1342 Seongnamdearo 13120, Republic of Korea; 2 ISC Co., Ltd., 215 Galmachi-ro, Jungwon-gu, Seongnam-si 13217, Gyeonggi-do, Republic of Korea

**Keywords:** semiconductor test sockets, rubber sockets, FeCo nanowires, Au coating, electrodeposition

## Abstract

The most promising approach for improving the electrical performance of connectors used in semiconductor test sockets involves increasing their electrical conductivity by incorporating one-dimensional (1D) conductive materials between zero-dimensional (0D) conductive materials. In this study, FeCo nanowires were synthesized by electroplating to prepare a material in which 1D materials could be magnetically aligned. Moreover, the nanowires were coated with highly conductive Au. The magnetization per unit mass of the synthesized FeCo and FeCo@Au nanowires was 167.2 and 13.9 emu/g, respectively. The electrical performance of rubber-based semiconductor connectors before and after the introduction of synthetic nanowires was compared, and it was found that the resistance decreased by 14%. The findings reported herein can be exploited to improve the conductivity of rubber-type semiconductor connectors, thereby facilitating the development of connectors using 0D and 1D materials.

## 1. Introduction

The demand for integrated-circuit (IC)-type semiconductor devices is accelerating to permit the fabrication of numerous electric and electronic components for use in fields such as information technology; biotechnology; automotive, industrial, medical, and defense industries; as well as in domestic and mobile communication devices, such as smartphones and tablets. Typically, semiconductor device packages are subjected to final electrical performance tests. However, the demand for high-bandwidth and small-form-factor packages is growing [1,2,3]. Therefore, semiconductor inspection—a crucial aspect of the semiconductor industry—must be targeted to provide non-defective devices [4,5].

The pogo-pin-type test socket is predominantly used for semiconductor inspection because of its high mechanical safety, rapid production feature, and low investment cost [6,7]. However, the types of semiconductors being used must change in accordance with the expanding semiconductor market, thereby necessitating changes in the semiconductor measurement environment. For example, high-band characteristics are required to reduce the semiconductor size for achieving miniaturization of electronic devices and developing miniaturized semiconductors that do not exhibit degraded performance [8,9,10]. To satisfy these requirements, the test sockets used to characterize the manufactured semiconductors must be diversified. However, the pogo-pin-type connectors have disadvantages in this regard, such as a high manufacturing cost, limited size setting, susceptibility to the measurement pressure, and material limitation in the high band. In contrast, rubber-type sockets can permit miniaturization because the particle size of the conductive powder can be adjusted, and the material constituting the connector can be applied at high bandwidths [2,5,11,12,13].

Typically, a rubber-type connector comprises a conductive material and insulating rubber (silicone), with the former being magnetically aligned during the fabrication of the socket. In this process, spherical conductive powder particles have a limited electrical conductivity because of their small contact areas. To solve this problem, the number of routes through which electricity can be conducted can be increased by creating an electrically conducting one-dimensional (1D) material between the zero-dimensional (0D) conductive powder particles [14,15,16].

The 1D material applied to the test socket is rapidly aligned by the magnet but should become demagnetized when the magnetic field is removed after alignment. To this end, fast magnetization and easy demagnetization characteristics are required. Materials with these properties are soft magnetic materials such as Fe-Si, Fe-Co, and Fe-Ni alloy, etc. Among these materials, the material with the best magnetization is FeCo [17,18,19]. However, in order to use it in the test socket, it is necessary to improve the electrical conductivity [18,20]. In order to improve electrical conductivity, it can be solved by coating highly conductive materials, such as Cu, Ag, and Au, on the surface [21,22,23,24].

Inspired by these approaches, materials with high conductivity and strong magnetism were synthesized in this study to lengthen the electron transport path between the conductive powder particles, which can minimize the electrochemical performance degradation of silicone-rubber-type semiconductor connectors. The magnetic material was readily synthesized using an electroplating method and then coated with a conductive material (Au) to impart conductivity. The resistance of the test socket that was prepared using the synthesized material was confirmed to decrease.

## 2. Materials and Methods

### 2.1. Formation of FeCo Nanowires and Au Coating

To synthesize FeCo nanowires (NWs), an electrolyte was prepared by dissolving FeSO_4_∙7H_2_O (0.15 M, SamChun, Pyeongtaek-si, Republic of Korea, 98%), CoCl_2_∙6H_2_O (0.2 M, Sigma-Aldrich, Darmstadt, Germany, 98%), and L-ascorbic acid (0.01 M, Sigma-Aldrich, Darmstadt, Germany, 99%) in deionized (DI) water. Anodic aluminum oxide (AAO, Whatman^TM^, Darmstadt, Germany, pore size: 200 nm, density: 10^11^/cm^2^) was coated with Au and then placed in the prepared electrolyte as the working electrode of a three-electrode system, which had Ag/AgCl and Pt as the reference and counter electrodes, respectively. To synthesize the FeCo NWs by electroplating, the reaction was conducted for 3 h by applying −1.1 V vs. Ag/AgCl and stirring the electrolyte at 80 rpm to ensure a constant ion distribution inside the electrolyte during the reaction. To remove the AAO electrode, in which the FeCo NWs were formed, the Au electrode part was initially removed and the leftover component was placed in a NaOH solution (3 M), which was then sonicated for 10 min. The obtained FeCo NWs were rinsed five times with DI water and then dried at 80 °C.

**Figure 1:**
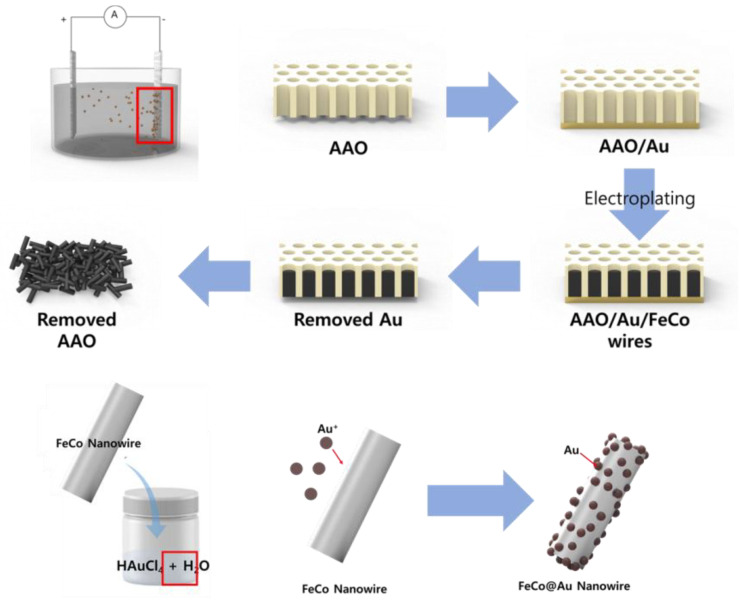
Schematic illustrating growth of FeCo nanowires (NWs) and their Au coating. (AAO: anodic aluminum oxide).

The electrolyte for electroless plating of Au on the FeCo NWs was prepared by dissolving HAuCl_4_ (6 mM, Sigma-Aldrich, Darmstadt, Germany, 49%) in DI water. HAuCl_4_ (6 mM) was added to 100 mL DI water and sonicated for 5 min. The FeCo NWs were added to the prepared Au electrolyte, dispersed using sonication and vortex-based methods (with another system left undispersed for comparison), and then reacted at 20 °C for 5 min. The resulting FeCo@Au NWs were collected by centrifugation, washed five times with DI water, and then dried overnight at 80 °C. Figure 1 shows the schematic of the synthesis sequence.

### 2.2. Characterization of FeCo@Au NWs

The surface morphology and composition of the FeCo NWs before and after the Au coating were assessed by scanning electron microscopy (SEM; Hitachi S-4300, Hitachi, Chiyoda, Japan) with an acceleration voltage of 15 kV and energy-dispersive X-ray spectroscopy (EDS; HORIBA 7021-H, HORIBA, Kyoto, Japan). The structure of the synthesized material was analyzed by X-ray diffractometry (XRD; SmartLab, Rigaku, Tokyo, Japan) with Cu Kα radiation and 2θ angle from 20° to 85° with a scanning speed 2°/min. Additionally, X-ray photoelectron spectroscopy (XPS; K-Alpha, Thermo Electron, Illinois, USA) was performed to confirm the changes in the sample surfaces before and after the synthesis. Data were acquired in constant energy analyzer mode with a narrow and survey scan pass energies of ΔE = 50 and 200 eV, respectively. The magnetism of the synthesized material was analyzed using a vibrating-sample magnetometer (VSM; VSM 7410, Lake Shore, OH, USA), where the external magnetic field was controlled to 25 kOe.

### 2.3. Fabrication of Semiconductor Test Connectors

Silicone mixed with magnetic particles was injected into a mold that contained pins for aligning them. A magnetic field was subsequently applied. The magnetic particles inside the silicone were aligned in the direction of the applied magnetic field, forming a conductive path through contact with the adjacent particles. A connector was manufactured by adding the coated NWs to the magnetic-particle-incorporated silicone at a weight ratio of 20:1; a NW-free connector was also fabricated for comparison. The resistance of each connector was measured by applying a voltage to its top and bottom. After placing the connector on a flat-bottomed Au substrate, voltage was applied by contacting the Au-coated probe to the top surface of the connector.

## 3. Results and Discussion

A three-electrode system containing AAO, Ag/AgCl, and Pt as the cathode, anode, and reference electrode, respectively, was used to synthesize the FeCo NWs. An acidic solution containing dissolved Fe^2+^ and Co^2+^ was used as the electrolytic solution. To determine the conditions for synthesizing the FeCo NW alloy, the reduction potential was estimated by linear sweep voltammetry (LSV), and a stable growth rate was simultaneously achieved at −1.1 V during the plating.

**Figure 2:**
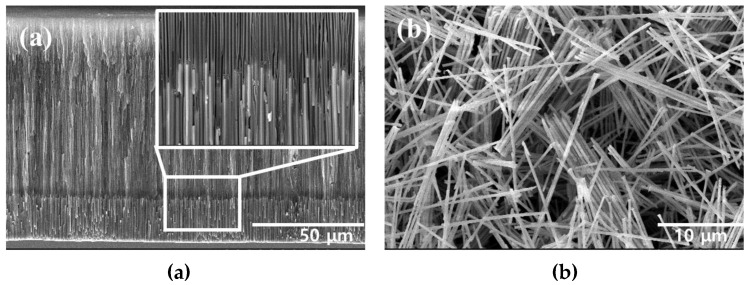
Scanning electron microscopy (SEM) images of (**a**) AAO/FeCo NWs (cross-section) and (**b**) pure FeCo NWs.

The FeCo NWs synthesized using AAO were examined by SEM. The cross-section of the AAO confirmed the growth of the FeCo NWs inside its pores (Figure 2a). Moreover, in the cross-sectional image of Figure S1a, the elemental distributions of AAO, Fe, and Co were confirmed to confirm that FeCo NWs were grown to a uniform length. Additionally, an SEM image of the FeCo NWs that were obtained after removing the AAO layer was acquired (Figure 2b), which indicated that the synthesized FeCo NWs were ~20-μm-long ~200-nm-thick cylinders with a smooth surface. EDX analysis was carried out to analyze the elemental content of FeCo NWs; the Fe:Co ratio was confirmed as 52:47 (Figure S1b).

**Figure 3:**
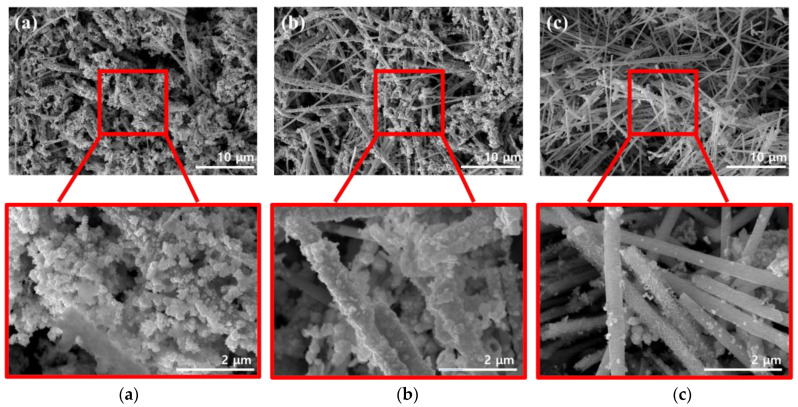
SEM images of FeCo NWs in different Au solutions for (**a**) sonication- and (**b**) vortex-based dispersion. (**c**) shows images of the undispersed mixture for comparison.

Figure 3 shows SEM images that were acquired while optimizing the dispersion method used for coating the conductive material, which helped impart electrical conductivity to the surface of the FeCo NWs. As the conductive material, Au, which has excellent electrical conductivity and high stability because it does not react with other materials, was used [25,26].

Additionally, the effects of the external energy required to activate the surface reaction had to be determined. Therefore, three methods were compared in this regard, with two involving dispersion and one used for comparison [27,28]. Sonication is a method that utilizes intense sound waves to disperse nanomaterials and organic materials [29,30,31]. In the vortex-based method, the solution is physically agitated to form a dispersion-enabling vortex [32,33]. To investigate the effects of each method, the reaction time and solution concentration were maintained constant. The SEM image of the FeCo@Au NWs synthesized by sonication (Figure 3a) reveals excessive formation of Au on the surface of the FeCo NWs, which was present as circular aggregates rather than as a coated film. The SEM image of the FeCo@Au NWs synthesized using a vortex (Figure 3b) suggests that Au was coated relatively evenly on the surface of the sample; this coating was more uniform that that obtained using the sonication method. The SEM image of the system realized when the FeCo NWs were added to the Au-containing solution and left undispersed (Figure 3c) confirmed that Au existed as particles or random shapes films on the surface of the FeCo NWs. In addition, the EDX analysis of the elemental distribution of Au, Fe, and Co confirmed that the distribution of Au was widely and evenly distributed in the other two methods (Figure S2). In contrast, the undispersed mixture confirmed the high surface exposure of FeCo NWs. Here, Au was present in the form of a few particles and a thin film, despite the synthesis being achieved under conditions identical to those employed in the other two methods. This was evidently due to the difference in the energy of the method used for the dispersion. Because more energy was transferred to the sample during sonication than in the vortex-based method, the deposition based on the conversion of Au^3+^ to Au^0^ was promoted, yielding overgrown Au. According to classical nuclear growth, the size of the generated nucleus increases with increasing free energy [34,35]. Therefore, a stronger energy than that in the vortex-based method was supplied during sonication, resulting in a high free energy; moreover, because the generated critical nucleus for additional growth was considerable in size, large Au particles were formed on the NW surfaces. Overall, these results indicate that the vortex-based method was optimal for coating Au on the FeCo NWs.

**Figure 4:**
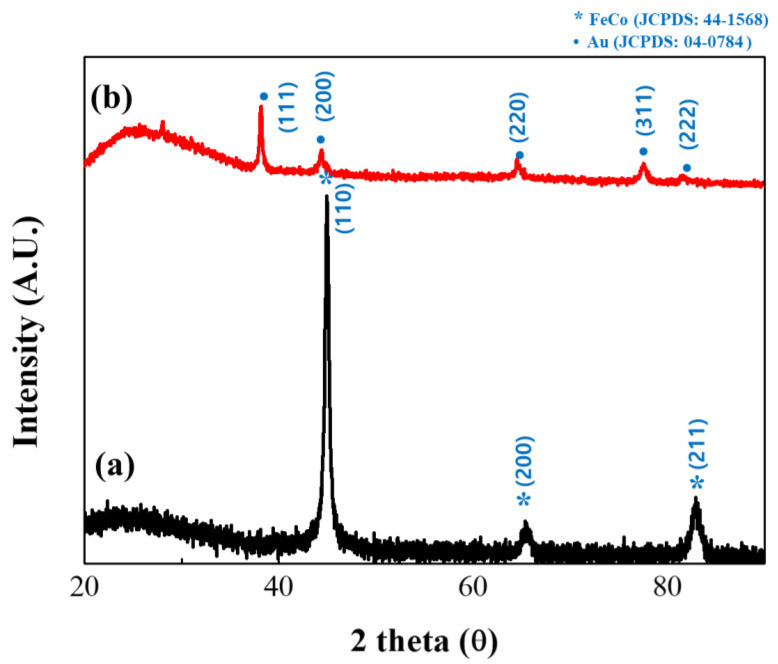
X-ray diffractometry (XRD) patterns of (a) uncoated and (b) Au-coated FeCo NWs (black and red profiles, respectively).

The structures of the synthesized FeCo and FeCo@Au NWs were subsequently analyzed by XRD (Figure 4). The uncoated FeCo NWs were found to have no other impurities, and the analysis revealed the body-centered cubic (bcc) structure of FeCo with intense peaks representing the (110), (200), and (211) crystal orientations as the three parts of the deflection peak. Additionally, the lattice parameter was estimated to be *a* = 2.849 Å, thereby confirming the existence of a similar structure to that of bulk FeCo [36,37,38]. The particle size was calculated using the Scherrer equation to be 14.37 nm [39].

The analysis of Au-coated FeCo NWs revealed the face-centered cubic (fcc) structure of Au indexed as (111), (200), (220), and (311). Additionally, in the XRD results of the Au-coated sample, since the angles at which the (200) and (220) planes of Au and (110) and (200) planes of FeCo are detected are similar, it seems that only Au overlapped [40,41,42,43].

**Figure 5:**
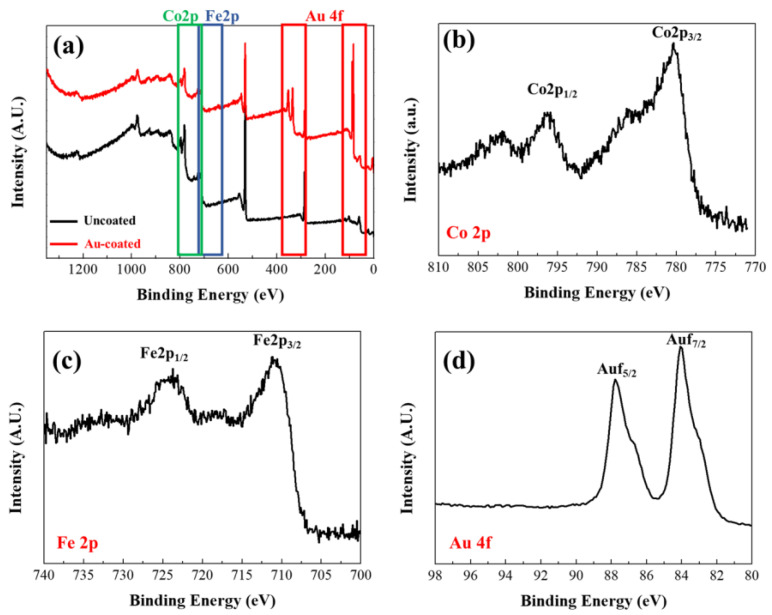
X-ray photoelectron spectroscopy (XPS) profiles of uncoated and Au-coated FeCo NWs: (**a**) survey, (**b**) Co 2p, (**c**) Fe 2p, and (**d**) Au 4f scans.

XPS measurements were subsequently conducted to confirm the surface characteristics of the synthesized FeCo NWs (Figure 5). An XPS survey scan (Figure 5a) was performed to obtain information on peaks according to the binding energy of the materials constituting the sample. The Au 4f peak appeared in the spectrum of the coated specimen in the region between 80 and 98 eV, thereby confirming the Au coating [44]. Co 2p XPS analysis revealed Co 2p_3/2_ and Co 2p_1/2_ peaks at 780.38 and 795.98 eV, respectively, and the Fe 2p XPS analysis revealed Fe 2p_3/2_ and Fe 2p_1/2_ peaks at 710.98 and 724.38 eV, respectively. These are presumed to correspond to atmospherically oxidized Co and Fe based on the binding energies of Co^2+^ and Fe^2+^, respectively. [38,45,46,47].

**Figure 6:**
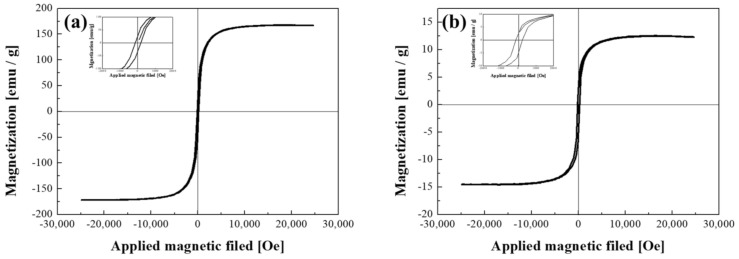
Hysteresis loops of (**a**) FeCo and (**b**) FeCo@Au NWs acquired using a vibrating-sample magnetometer (VSM).

The FeCo NWs were then prepared to form a conductive path by placing them between Au-coated magnetic particles and silicone for semiconductor socket inspection. A certain level of magnetic force was required to enable this investigation. Thus, the magnetic properties of the synthesized material were analyzed using a VSM (Figure 6). The magnetization per unit mass (*M*_s_) of the uncoated FeCo NWs was 167.2 emu/g, which is similar to the results obtained in several previous studies (Table 1). Moreover, the corresponding *M*–*H* curve exhibited typical soft magnetic characteristics, as observed previously [48,49,50,51,52,53,54,55,56]. The low *M*_s_ values of the NWs or tubes listed in Table 1 were presumably caused by the dimensions of the synthetic material [57,58]. After the Au coating, the sample exhibited a magnetization per unit mass of 13.9 emu/g, which was considerably lower than that of the uncoated FeCo NWs. This is because the weight of the sample for VSM analysis includes the magnetic material and the coating material, which is due to the increase in the content of the non-magnetic material [59,60,61]. However, the coercivity (*H*_c_) values were found to be similar. The coercivity is a performance-related parameter that is closely related to the crystallite size of a magnetic material and can determine the magnetic interactions between crystallites and microstructural changes [62,63,64]. The coercivity of the FeCo and FeCo@Au NWs were determined to be 122.2 and 151.3 Oe, respectively, indicating the lack of Au-coating-induced microstructural changes in the FeCo NWs. *H*_c_ slightly increased after the plating because it varies according to the vertical and horizontal directions of the applied magnetic field for wire-shaped magnetic materials [42], that is, because of the difference in the alignment of the FeCo@Au NWs.

Consequently, it was confirmed that the magnetization per unit mass measured after Au electroless plating varied as the Au coating layer was formed. Nevertheless, the strategy reported herein can be applied to silicone rubber for semiconductor inspection because the magnetic force can be maintained after the Au coating.

**Figure 7:**
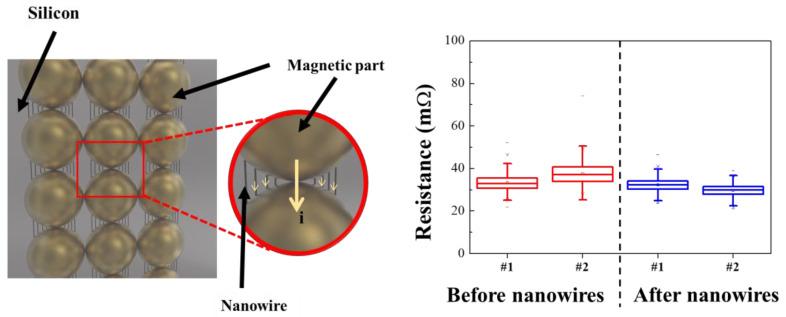
Illustration of test sockets with the FeCo@Au NWs and their resistance data.

A prototype sample was then fabricated for applying the synthesized FeCo@Au NWs to a socket for semiconductor testing (Figure 7). An empty space was present between the conductive powder particles prior to introducing the NWs. Because this space is occupied by rubber—an insulator—the movement of electrons, which causes electron loss and increases the internal resistance. In contrast, the presence of the FeCo NWs in the space between the conductive metals reduces the internal resistance by increasing the electron movement pathway and facilitating electron motion.

The pristine and NW-containing samples exhibited resistances of 34.5 and 31 mΩ, respectively, indicating that the resistance was reduced by approximately 14%. The resistance obtained by introducing NWs showed better characteristics than conventional IC rubber-type test sockets [65,66]. Therefore, the electrical properties of a socket prepared using the synthesized FeCo@Au NWs for semiconductor analysis was improved, and the industrial application potential of this approach was confirmed through stabilized material synthesis.

## 4. Conclusions

In this study, a method for improving the electrical properties and performance of sockets used for semiconductor inspection was investigated by constructing narrow electrical paths between the conductive powder particles. Compact electrical routes can be realized by exploiting both magnetic and conductive properties. The magnetic material was synthesized by electroplating FeCo NWs using a three-electrode method, and three dispersion methods were investigated to achieve uniform Au plating. The vortex-based method resulted in the formation of the most uniform coating layer among the investigated approaches. The magnetization per unit mass of the FeCo NW synthesized by the electroplating was 167.2 emu/g, which, after gold plating, was 13.9 emu/g. The coercive force did not change significantly, confirming that there was no change in the magnetic properties. In addition, the resistance of the socket before and after application of the synthesized FeCo@Au NWs was 34.5 and 31 mΩ, respectively. The synthesized FeCo@Au NWs were confirmed to decrease the resistance of a socket via the construction of electrically conductive paths between the conductive powder particles.

## 5. Patents

The results obtained in this study have been patented in the Korea (application no.: 10-2022-0049406).

## Figures and Tables

**Table 1:** Magnetic properties of previously reported FeCo alloy specimens.

Method	Morphology	Fe:Co [at.%]	*M*_s_ [emu/g]	Refs.
**Chemical method (such as co-precipitation and chemical reduction)**	Powder	50:50	207	[45]
	Nanocubes	50:50	211.9	[46]
	Hollow spheres	48:51	169.35	[47]
	NWs	60:40	92	[48]
**Laser additive manufacturing (such as laser-engineered net shaping)**	Film	40:60	201.7	[50]
	Film	30:70	199.3	
**Electrodeposition**	Nanotubes	50:50	100	[51]
	NWs	49:51	176	[52]
**Electrodeposition** **(Present study)**	NWs	46:54	167.2	

## Data Availability

Not applicable.

## Supplementary Materials

The following are available online at https://www.mdpi.com/article/10.3390/ma16010381/s1, Figure S1: SEM and EDX images of (a) AAO/FeCo NWs (cross-section) and (b) pure FeCo NWs and Figure S2: SEM and EDX image of FeCo NWs in different Au solutions for (a) sonication- and (b) vortex-based dispersion. (c) Images of the undispersed mixture for comparison.
