# Supplementary material for: Fabrication and Characteristics of a Conductive FeCo@Au Nanowire Alloy for Semiconductor Test Socket Connectors
Source: Materials (Basel). 2022 Dec 30;16(1):381. doi: 10.3390/ma16010381 (PMC9821946; doi:10.3390/ma16010381)
Supplement: Supplementary file 1 [file materials-16-00381-s001.zip › materials-2112336-supplementary.pdf]

Supplementary Materials

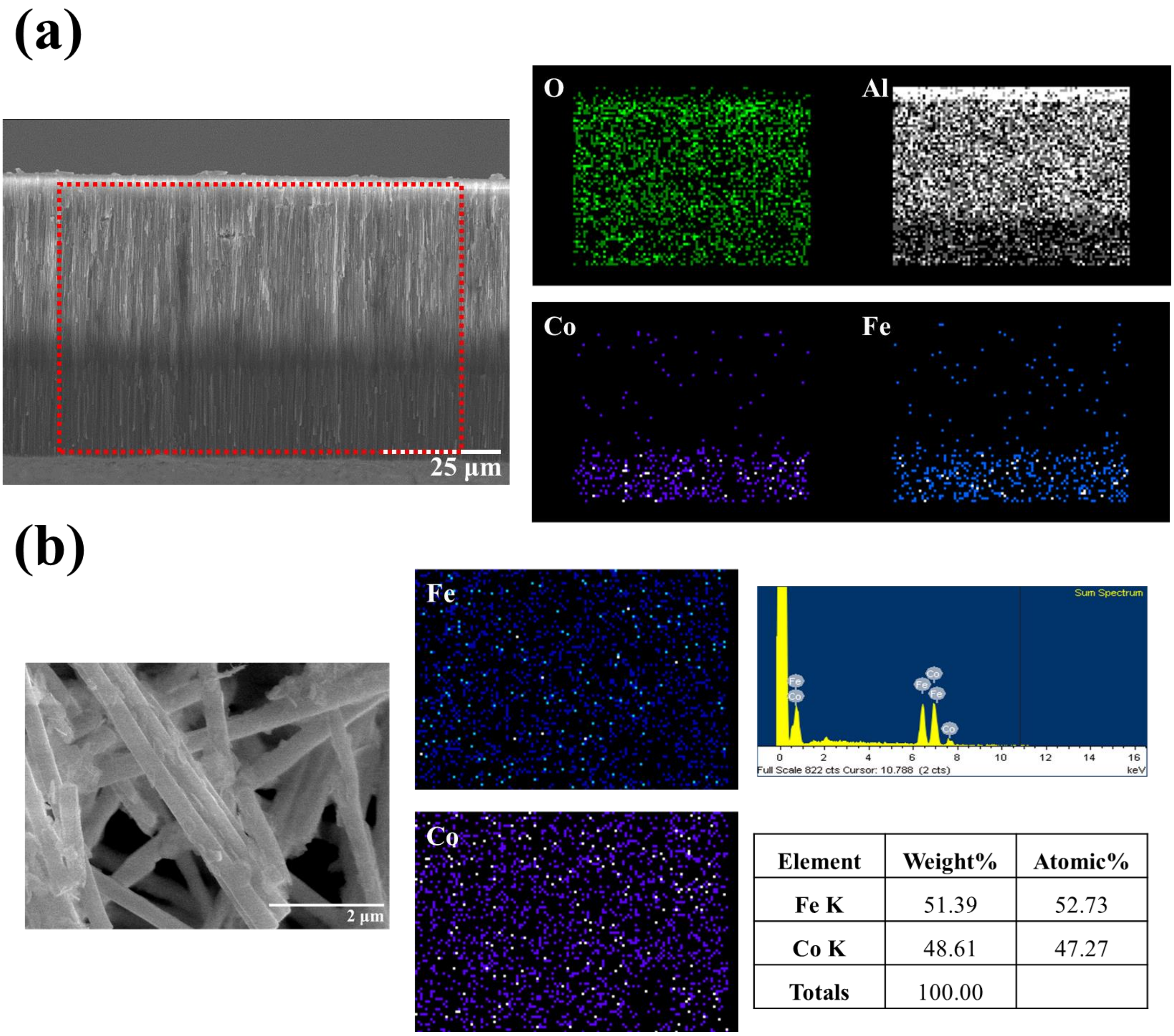

Element	Weight%	Atomic%
Fe K	51.39	52.73
Co K	48.61	47.27
Totals	100.00	

Figure S1. SEM and EDX images of (**a**) AAO/FeCo NWs (cross-section) and (**b**) pure FeCo NWs.

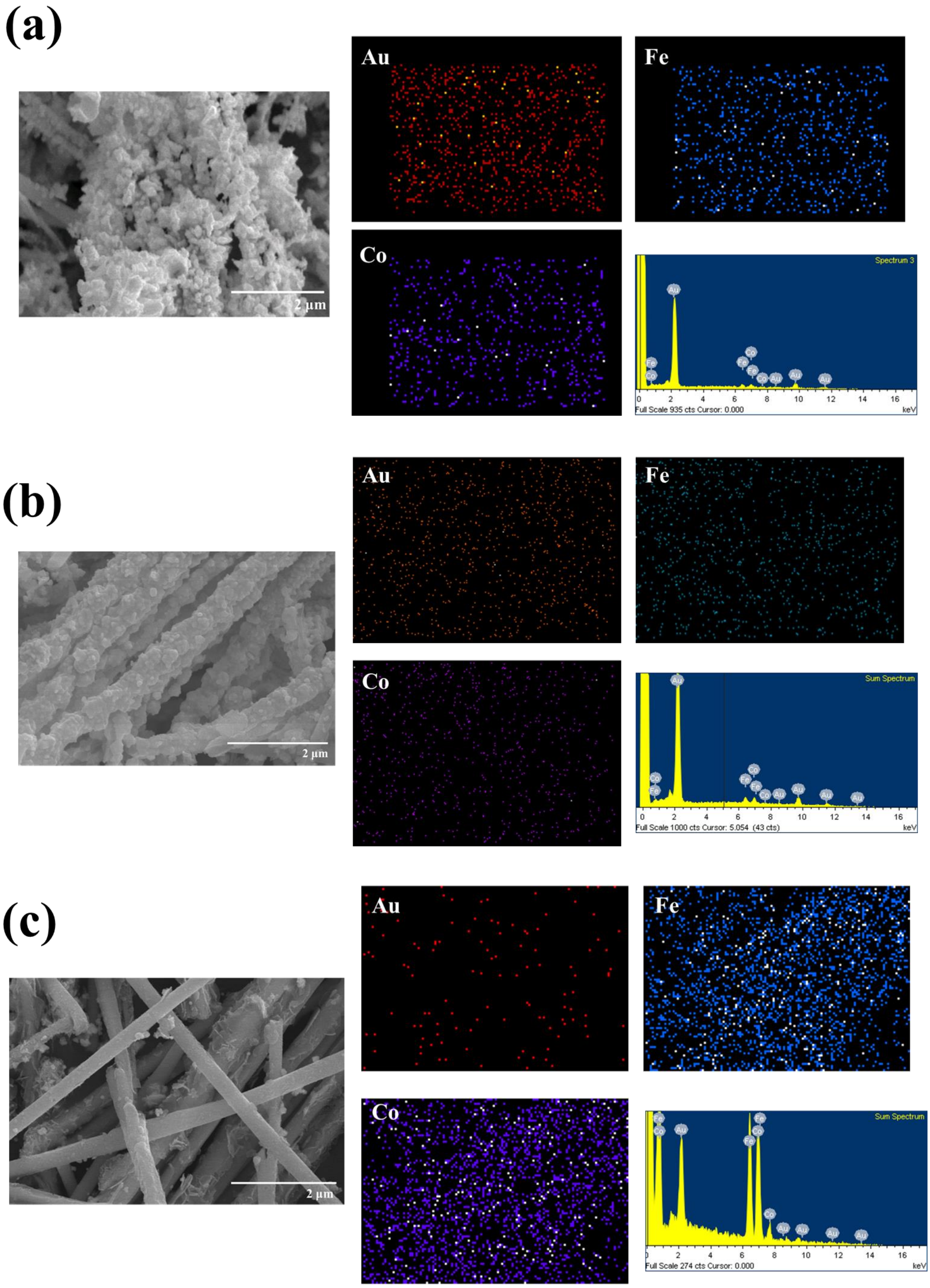

Figure S2. SEM and EDX image of FeCo NWs in different Au solutions for (**a**) sonication- and (**b**) vortex-based dispersion. (**c**) Images of the undispersed mixture for comparison.
